# Reporting and dealing with missing quality of life data in RCTs: has the picture changed in the last decade?

**DOI:** 10.1007/s11136-016-1411-6

**Published:** 2016-09-20

**Authors:** S. Fielding, A. Ogbuagu, S. Sivasubramaniam, G. MacLennan, C. R. Ramsay

**Affiliations:** 1 Institute of Applied Health Sciences, University of Aberdeen, Polwarth Building, Foresterhill, Aberdeen, AB25 2ZD UK; 2 Health Services Research Unit, Institute of Applied Health Sciences, University of Aberdeen, Aberdeen, UK

**Keywords:** Missing data, Quality of life, Randomised controlled trial, Imputation

## Abstract

**Purpose:**

Missing data are a major problem in the analysis of data from randomised trials affecting power and potentially producing biased treatment effects. Specifically focussing on quality of life outcomes, we aimed to report the amount of missing data, whether imputation was used and what methods and was the missing mechanism discussed from four leading medical journals and compare the picture to our previous review nearly a decade ago.

**Methods:**

A random selection (50 %) of all RCTS published during 2013–2014 in BMJ, JAMA, Lancet and NEJM was obtained. RCTs reported in research letters, cluster RCTs, non-randomised designs, review articles and meta-analysis were excluded.

**Results:**

We included 87 RCTs in the review of which 35 % the amount of missing primary QoL data was unclear, 31 (36 %) used imputation. Only 23 % discussed the missing data mechanism. Nearly half used complete case analysis. Reporting was more unclear for secondary QoL outcomes. Compared to the previous review, multiple imputation was used more prominently but mainly in sensitivity analysis.

**Conclusions:**

Inadequate reporting and handling of missing QoL data in RCTs are still an issue. There is a large gap between statistical methods research relating to missing data and the use of the methods in applications. A sensitivity analysis should be undertaken to explore the sensitivity of the main results to different missing data assumptions. Medical journals can help to improve the situation by requiring higher standards of reporting and analytical methods to deal with missing data, and by issuing guidance to authors on expected standard.

**Electronic supplementary material:**

The online version of this article (doi:10.1007/s11136-016-1411-6) contains supplementary material, which is available to authorized users.

## Introduction

The randomised controlled trial (RCT) is often regarded the gold standard study design for evaluating healthcare interventions but can be prone to missing outcome data. At best missing data reduce the sample size and power of an RCT and at worst could bias results. Patient-reported quality of life (QoL) outcomes are essential to inform decisions about best available treatments. Missing data are problematic for any outcome, but with QoL outcomes missing data are often informative. Ignoring missing data may bias estimates of treatment effects. The literature is extensive on the consequences of ignoring missing data and methods to deal with it [1–4]. Guidelines do exist, but the question remains as to whether these guidelines are being followed [5]. Understanding the mechanism of the missing data is not a new concept. Little and Rubin defined three missing data mechanisms: missing completely at random (MCAR), missing at random (MAR) and missing not at random (MNAR) [6]. It is difficult to prove data are MNAR because by definition the data are missing, but it is possible to differentiate between MCAR and MAR [7]. Methods of analysis depend on the missing data assumption; however, published reports of RCTs rarely justify the method choice [1].

The simplest method for handling missing data is to ignore it and use complete case analysis (CCA). In CCA, all participants with missing data are excluded. CCA is, however, suitable only if data are MCAR and the missing data proportion is small [4]. CCA produces biased estimates if data are MAR or MNAR. The use of sensitivity analysis to test the impact of different missing data assumptions is useful [5]. This may include imputation which can be something as simple as last value carried forward (LVCF), mean imputation or alternatively the more complex multiple imputation. The latter allows for more uncertainty by creating multiple imputed values and then using Rubin’s rules to combine the results [6–8]. In situations where data are collected repeatedly, the use of a repeated measures approach with the MAR assumption may be sensible. For MNAR, more sophisticated methods such as pattern mixture models may be useful, but these are less accessible to the average researcher [9, 10].

In 2008, we published a review of RCTs appearing in four medical journals during 2005–2006 and assessed the use of imputation to overcome missing QoL outcome data [1]. The aim here is to undertake a similar review of articles published in the same four journals during 2013–2014 to ascertain whether the picture has changed in light of that review and other similar recent literature. Specifically, we aim to report the amount of missing data, whether imputation was used and what methods and was the missing mechanism discussed. We will compare the findings to those of our previous review.

## Methods

### The search

**Fig. 1:**
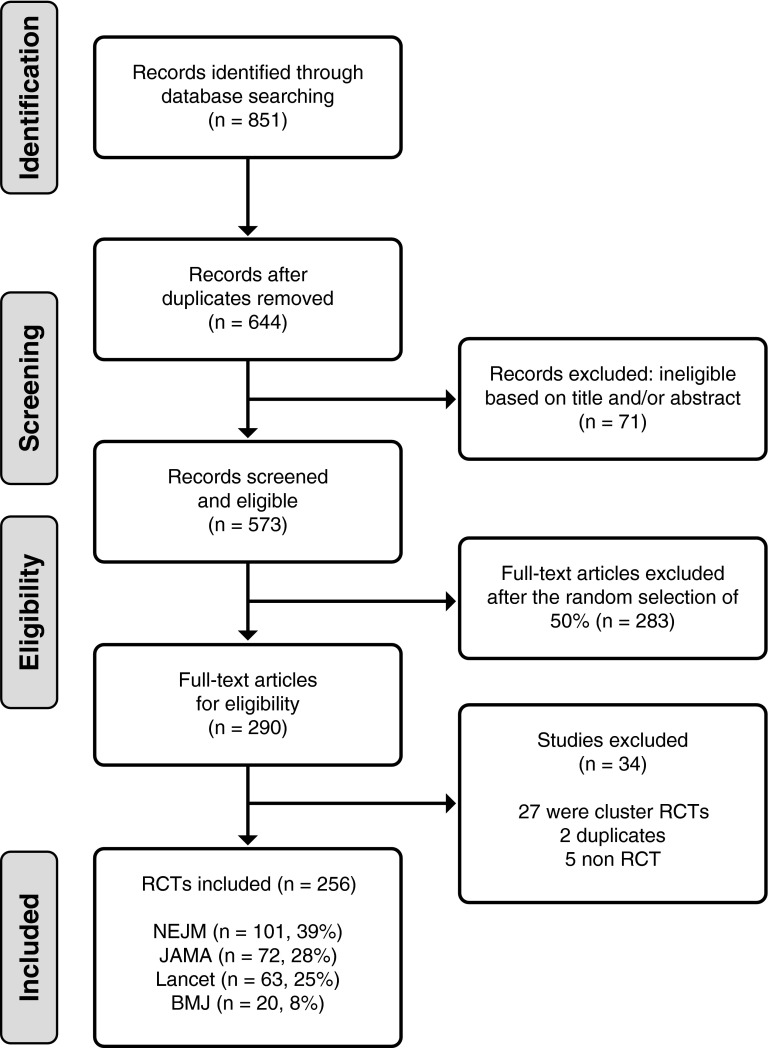
PRISMA flow diagram for study inclusion

We searched MEDLINE and Embase for RCTs published during 2013 and 2014 in four leading medical journals: the British Medical Journal (BMJ), the Journal of American Medical Association (JAMA), the New England Journal of Medicine (NEJM) and the Lancet. These four journals were chosen as they are often the first choice for publication of RCT results. Inclusion criteria were any RCT published in one of the four target journals (BMJ, JAMA, NEJM, Lancet) during 2013 or 2014. RCTs reported in research letters, cluster RCTs (as statistical issues differ as randomisation does not occur at individual level), non-randomised designs, review articles and meta-analyses were excluded. Figure 1 details the selection of studies to be included. The search identified 851 trials, and after removal of duplicates and ineligible studies on title and abstract, we had 573 for potential inclusion. A random sample of 50 % was taken forwards to full text review (*N* = 290). No quality assurance was undertaken as we were not interested in the results of the studies. Abstract review was undertaken by one researcher (AO) after an initial double screening of 25 articles by a second reviewer (SF or SS). In all 25 cases, agreement on inclusion was agreed, so thereafter, only one reviewer undertook the abstract screening, with any uncertainties about inclusion discussed with a second reviewer (SF).

### Data extraction and analysis methods

We extracted basic information about each RCT including: amount of missing data, type of QoL outcome, was imputation used (if so what method), number of intervention arms, number randomised, whether the mechanism of missing data was discussed, analysis method used and if sensitivity analysis was undertaken. Extraction of information was restricted to the main QoL outcome (whether that be a primary or secondary outcome within the RCT). One researcher (AO) carried out data extraction, after an initial pilot of double abstraction (SF or SS) of 50 articles showed consistency between reviewers. Any queries on singly abstracted articles were resolved with a second reviewer (SF).Information collected was then entered into an IBM SPSS Statistics 22 database. Frequencies and percentages for characteristics of included RCTs were shown. Continuity corrected Chi-squared tests were used to compare outcomes between our previous review [1] and this current review. On completion of analysis, SF double-checked the key information from each included article to ensure accuracy. A few corrections were made, and analysis rerun to produce the results contained here.

## Results

### Search results

Figure 1 shows the searching process yielding 256 RCTs for the review. The majority of RCTs (*n* = 195, 76 %) were two-arm studies, with a further 36 (14 %) three arms and the remaining 21 (8 %) four or more arms. Most of the studies were of parallel design (94 %) with four studies (2 %) using a crossover design and 11 (4 %) a factorial design. Within the 256 RCTs, 91 (36 %) said they included a QoL outcome; however, in four RCTs the analysis of the QoL was not reported and these were excluded. We included 87 RCTs in which at least one QoL outcome was reported in 34 (39 %) as primary outcome, 57 (61 %) as secondary outcome).

### Description of missing data

Table 1 describes the amount of missing data. Four RCTs reported no missing data, and it was unclear for 30 (35 %). In the 52 RCTs not using imputation, 17 (33 %) had an unclear amount of missing data and 18 had less than 10 % missing. In the 31 RCTs that used imputation, for ten (29 %) the amount of missing data was unclear and 13 (39 %) had less than 10 % missing, 7 (21 %) had between 10 and 20 % missing with only one having more than 20 % missing. A comparison between the amount missing between primary QoL and secondary QoL found that it was unclear for 12 % of primary QoL but 49 % of secondary QoL (data not shown).

**Table 1:** Distribution of level of missing QoL data in RCTs

Proportion missing	No imputation (*N* = 52)	Imputation (*N* = 31)	Unclear (*N* = 4)	Total (*N* = 87)
None	4	0	0	4 (5 %)
<5 %	9	6	0	15 (17 %)
5–10 %	9	7	0	16 (18 %)
11–20 %	6	7	0	13 (15 %)
>20 %	7	1	1	9 (10 %)
Unclear	17	10	3	30 (35 %)

The missing data mechanism was discussed in 19/83 (23 %) of eligible RCTs, as four had no missing data. However, an indication of which mechanism was only provided for 11 (eight MAR, two MCAR, one MNAR). The remaining 64/83 (77 %) RCTs had no discussion of the missing data mechanism at all.

### Quality of life measures used

A variety of QoL measures were used; the main QoL outcome in 34 (39 %) RCTs was a generic measure such as EQ-5D or SF-12/SF-36. The remaining 61 % were disease specific measures covering a wide range of disease areas. Examples included: QLQ-C30, PDQ39, Seattle Angina Questionnaire, Ankylosing Spondylitis QoL, DEMOQOL (QoL for people with dementia), Western Ontario and McMaster Universities Osteoarthritis Index (WOMAC), Function assessment of Chronic Illness therapy-fatigue (FACIT-F), Visual Function questionnaire (VFQ-25) and Brief Pain Inventory.

### Imputation methods

Table 2 describes basic characteristics of the 87 included RCTs that contained a QOL outcome. The QoL outcome was continuous in 63 (72 %) RCTs and analysed at a single endpoint in 57 RCTs (66 %). In total, 18 (21 %) RCTs used imputation within their primary analysis, and a further 13 (15 %) RCTs used it within a sensitivity analysis resulting in 31/87 (36 %) included RCTs using some form of imputation.

**Table 2:** Description of the 87 RCTs with a QoL outcome included in the review

		Primary QoL (*N* = 34)	Secondary QoL (*N* = 53)	Total (*N* = 87)
Journal	BMJ	6 (18 %)	6 (11 %)	12 (14 %)
	NEJM	11 (32 %)	18 (34 %)	29 (33 %)
	JAMA	4 (12 %)	14 (26 %)	18 (21 %)
	Lancet	13 (38 %)	15 (28 %)	28 (32 %)
Number of intervention arms	2	27 (79 %)	49 (92 %)	76 (87 %)
	3	5 (15 %)	2 (4 %)	7 (8 %)
	4+	2 (6 %)	2 (4 %)	4 (5 %)
QoL outcome	Binary	7 (21 %)	2 (4 %)	9 (10 %)
	Continuous	20 (59 %)	43 (81 %)	63 (72 %)
	Categorical	7 (21 %)	6 (11 %)	13 (15 %)
	Count	0 (0 %)	1 (2 %)	1 (1 %)
	Other	0 (0 %)	1 (2 %)	1 (1 %)
Endpoint	Single	18 (53 %)	39 (74 %)	57 (66 %)
	Repeated	16 (47 %)	14 (26 %)	30 (34 %)
Analysis	Complete case	21 (62 %)	35 (66 %)	56 (64 %)
	Imputation	2 (6 %)	10 (19 %)	12 (14 %)
	Repeated measures	11 (32 %)	8 (15 %)	18 (21 %)
Imputation used	Yes in primary analysis	7 (21 %)	11 (21 %)	18 (21 %)
	Yes as sensitivity	8 (24 %)	5 (9 %)	13 (15 %)
	No	19 (56 %)	33 (62 %)	52 (60 %)
	Unclear	0 (0 %)	4 (8 %)	4 (5 %)

For the 18 RCTs employing imputation within their primary analysis, four used last value carried forwards (LVCF); five used worst case scenario; one mean value imputation; seven used multiple imputation (two chained equations, one Markov chain Monte Carlo (MCMC) and unclear for four). Of the 13 RCTs where imputation was used as a sensitivity analysis, twelve used multiple imputation (with only one indicating which method, predictive mean match) and one used worst case scenario.

### Analysis methods

The primary analysis strategy for the QoL outcome was a complete case analysis for 42/87 RCTs (48 %) and 33 (38 %) a repeated measures approach, the remainder using imputation (Table 2). Twelve of the 33 RCTs which used a repeated measures approach did so with imputation undertaken as well (six within primary analysis and six within sensitivity). The main complete case analysis was linear regression/analysis of covariance as the majority of QoL outcomes were continuous.

### Comparison of results to the previous review

Table 3 shows a main comparison of the findings from our previous review [1] and those we have identified in the current review. We can see there has been an increase in the proportion of RCTs reporting at least one QoL outcome (21.4–34.0 %, *p* = 0.001). This increase in the use of QoL outcomes is not surprising given the recent emphasis on patient-reported outcomes within RCTs.

**Table 3:** Comparison of 2005–2006 and 2013–2014 RCTs

	2005–2006	2013–2014
RCTs reviewed	285	256
Include QoL outcome	61 (21.4 %)	87 (34.0 %)
Imputation	19/61 (31.1 %)	31/87 (35.6 %)
Use of MI	1	20
Missing data discussed		
Proportion of missing data	*N* = 61	*N* = 87
None	9.8	4.6
<10 %	34.4	35.6
11–20 %	18.0	14.9
>20 %	18.0	10.3
Unclear	19.7	34.5

Despite a slight increase in the proportion of RCTs using imputation, the difference was not significant (*p* = 0.696). Imputation methods used previously focussed around simple imputation, whereas now we have seen a shift and a higher number have used multiple imputation (20/31 using MI here compared to only 1/19 in the previous review, *p* < 0.001). The amount of missing data was broadly similar.

## Discussion

We have found that QoL outcomes are increasingly being used as outcomes within RCTs, and missing data are still a major problem. In particular, compared to 2005 review, there is less clarity on the description of missing data for secondary outcomes. Researchers favoured simple but potentially biased methods such as last value carried forwards or worse value imputation in their main analysis. Multiple imputation was used more frequently than in our previous review but mainly for sensitivity analyses. Discussion of the missing data mechanism was limited, so it is unclear whether the imputation methods used were appropriate as insufficient information is provided by authors. Although researchers appear more aware, they should account for missing data their primary analyses were still often based on complete case analysis.

Our findings are specific to QoL outcomes; however, what we have found is mirrored in more general reviews such as Wood et al. [2] and Bell et al. [3]. In both of these, they found complete case was the most common analysis strategy, and simple imputation used more often than multiple imputation despite recommendations against it. Like us, they also found that reporting of the assumptions around the missing data mechanism was limited.

In our previous review, we recommended clearer reporting of missing data, the impact of missing data discussed and sensitivity analyses reported. It is clear from our current review and others that this is still not being undertaken appropriately in the majority of studies [3, 11]. In situations of longitudinal data, many studies did not exploit the repeated nature of the data in the analysis, and participants were excluded within a complete case because they did not have data for the final endpoint. In a mixed model, for example, these participants would have been included if they had provided at least one QoL assessment at some other time. Where multiple imputation was used, it was not always clear what the imputation model contained. This should be reported, and at the very least it should contain any variables used in the randomisation process and those used in the analysis model [12] It would be advantageous to use other auxiliary variables which may improve the imputation, e.g., using a previous QoL score to impute one that is missing [9, 12].

The four journals covered in this review are four of the top five medical journals with respect to impact factors. Using only these four could be considered a potential limitation. As these journals are considered the pinnacle of RCT publication, one might argue they should have the best reporting standards; however, this is potentially not the case. While we have limited our research to these four journals, the findings will be directly applicable to any journal which contains articles reporting the results of RCTs. The four journals provide very few guidelines on how to report missing data. The Lancet and BMJ direct authors to adhere to the CONSORT guidelines [13], but JAMA and NEJM make no such recommendations. The CONSORT checklist for reporting RCTs indicates information should be provided on loss to follow-up and the reason for exclusion, along with numbers randomised and analysed. Reporting of missing data is not mentioned directly in the statement. We compared the type of analysis, whether or not missing data mechanism was discussed, imputation use and missing data proportion between those articles published in BMJ/Lancet versus NEJM/JAMA to ascertain whether this recommendation of using the CONSORT made a difference (supplementary Table 1). No differences were found, indicating that the missing data reporting was no better in those journals who directed authors towards the CONSORT statement. While we cannot conclude a causal effect, this suggests the CONSORT is not adequate in providing guidelines to authors on how to report missing data for their RCTs.

However, more recently, Calvert et al. [14] have reported an extension to CONSORT for patient-reported outcomes, namely CONSORT PRO. In the CONOSRT PRO, statement P12a says ‘Statistical approaches for dealing with missing data are explicitly stated’. This is an important addition as it directly asks authors whether they have stated how they have dealt with missing data of patient-reported outcomes. This guidance was only published in 2013, so perhaps too recent to be reflected in the articles we have included in our review.

If authors are directed to the CONSORT checklist (and not CONSORT PRO), reporting of missing data is not explicitly detailed. Therefore, it is perhaps not surprising that authors still tend to favour complete case analysis. The reasons for this are unknown; however, from our own experience this may be due to word count issues or indeed some of the authors of this paper have tried to include an MI model into a manuscript, but the journal has insisted it is put in the supplement. The European Medicine Agency in 2010 recommended that studies provide ‘a detailed description of pre-planned methods used for handing missing data, and amendment of that plan and justification for those amendments should be included in the study report’ [15]. This raises the question to educate not just authors in how best to report missing data but also the journal editors who are deciding what articles are published.

QoL outcomes from RCTs increasingly inform cost-effectiveness analyses used by policy makers to decide on the allocation of resources. For example in the UK, the National Institute for Health and Care Excellence (NICE) relies on trial data (both clinical and cost-effectiveness) to make decisions about what treatments/interventions to use in the NHS. Therefore, it is imperative that findings from RCTs are robust that includes assessing the impact of and dealing with any missing data. Ignoring missing data may result in misleading findings through biased and imprecise estimates of treatment effects.

## Conclusions

Inadequate reporting and handling of missing QoL data in RCTs are still an issue. As Bell et al. concluded that there is an apparent large gap between statistical methods research relating to missing data and the use of the methods in applications, i.e., RCTs [3]. Medical journals could help to improve the situation by requiring higher standards of reporting and analytical methods to deal with missing data, and by issuing guidance to authors on expected standard. CONSORT is the gold standard of reporting RCTs, and journals direct authors towards CONSORT, but perhaps the checklist for RCTs needs to be more explicit about what information should be reported in regard to missing data. Journals should now direct authors to CONSORT PRO so if they have included a patient-reported outcome it is reported adequately. In addition, the missing data statement for QoL outcomes may encourage more information to be provided by authors on the other outcomes in their trials. Researchers themselves need to take responsibility for reporting detail in their own manuscripts and if reviewing manuscripts for journals or colleagues, ensure that they raise this issue where detail is lacking.

## Electronic supplementary material

Below is the link to the electronic supplementary material.

Supplementary material 1 (DOCX 71 kb)
