# Supplementary material for: Reporting and dealing with missing quality of life data in RCTs: has the picture changed in the last decade?
Source: Qual Life Res. 2016 Sep 20;25(12):2977–83. doi: 10.1007/s11136-016-1411-6 (PMC5102945; doi:10.1007/s11136-016-1411-6)
Supplement: Supplementary file 1 [file 11136_2016_1411_MOESM1_ESM.docx]

Supplementary table: Comparison between articles published in BMJ/Lancet versus JAMA/NEJM

		CONSORT		
		No<br>BMJ/Lancet<br>(n = 57)	Yes<br>JAMA/NEJM<br>(n = 30)	p-value
QoL outcome	Primary	24 (42.1)	10 (33.3)	0.571
	Secondary	33 (57.9)	20 (66.7)	
Analysis	Complete case	27 (47.4%)	15 (50%)	0.757
	imputation	7 (12.3%)	5 (16.7%)	
	repeated measures	23 (40.4%	10 (33.3%)	
Missing data mechanism discussed	No	45 (78.9%)	23 (76.7%)	>0.99
	Yes	12 (21.1%)	7 (23.3%)	
Imputation	No	33 (57.9%)	19 (63.3%)	0.915
	Yes in primary analysis	12 (21.1%)	6 (20.0%)	
	Yes in sensitivity	9 (15.8%)	4 (13.3%)	
	Unclear	3 (5.3%)	1 (3.3%)	
Missing data proportion	None	4 (10.5%)	0 (0%)	0.435
	<5%	11 (28.9%)	4 (21.1%)	
	5-10%	11 (28.9%)	5 (26.3%)	
	10-20%	7 (18.4%)	6 (31.6%)	
	>20%	5 (13.2%)	4 (21.1%)	
